# BHARAT: An Integrated Big Data Analytic Model for Early Diagnostic Biomarker of Alzheimer's Disease

**DOI:** 10.3389/fneur.2019.00009

**Published:** 2019-02-08

**Authors:** Ankita Sharma, Deepika Shukla, Tripti Goel, Pravat Kumar Mandal

**Affiliations:** ^1^Neuroimaging and Neurospectroscopy Laboratory (NINS), National Brain Research Centre, Gurgaon, India; ^2^Florey Institute of Neuroscience and Mental Health, University of Melbourne Medical School Campus, Melbourne, VIC, Australia

**Keywords:** big data framework, Hadoop, Alzheimer's disease, glutathione depletion, structural MRI, MRS, neuropsychological score, ensemble-based classification

## Abstract

Alzheimer's disease (AD) is a devastating neurodegenerative disorder affecting millions of people worldwide. Progressive and relentless efforts are being made for therapeutic development by way of advancing understanding of non-invasive imaging modalities for the causal molecular process of AD. We present a Hadoop-based big data framework integrating non-invasive magnetic resonance imaging (MRI), MR spectroscopy (MRS) as well as neuropsychological test outcomes to identify early diagnostic biomarkers of AD. This big data framework for AD incorporates the three “V”s (volume, variety, velocity) with advanced data mining, machine learning, and statistical modeling algorithms. A large *volume* of longitudinal information from non-invasive imaging modalities with colligated parametric *variety* and speed for both data acquisition and processing as *velocity* complete the fundamental requirements of this big data framework for early AD diagnosis. Brain structural, neurochemical, and behavioral features are extracted from MRI, MRS, and neuropsychological scores, respectively. Subsequently, feature selection and ensemble-based classification are proposed and their outputs are fused based on the combination rule for final accurate classification and validation from clinicians. A multi-modality-based decision framework (BHARAT) for classification of early AD will be immensely helpful.

## Introduction

Alzheimer's disease (AD) is a neurodegenerative disorder affecting elderly people and no cure is available to date. Alzheimer's disease is evidenced by cognitive decline and colligated behavioral disruption affecting activities of daily life (1–3). The actual cause of AD is still unknown, but amyloid beta peptide deposition and oxidative stress, specifically depletion of antioxidant glutathione in the hippocampal region (4, 5), are believed to play important roles in AD pathogenesis. Multi-modal imaging techniques such as MRI, MRS, functional MRI (fMRI), and positron emission tomography (PET), are being used extensively to identify early diagnostic biomarkers for AD. The behavioral information derived from various neuropsychological tests such as clinical dementia rating (CDR) (6), mini-mental state examination (MMSE) (6), the functional assessment questionnaire (FAQ) (7), the Hachinski ischemic score (HIS) (8), the geriatric depression scale—short form (GDS-SF) (9), and trail-making test A and B (TMT-A and TMT-B) (10) are useful to aid in AD diagnosis. The heterogeneous and diverse data generated worldwide from imaging, spectroscopy, and neuropsychology necessitate a common platform for a coherent multi-modal data processing and analysis scheme for the identification of distinctive diagnostic features specific to AD.

To date, machine learning (ML)-based techniques are being used with uni-modal data (structural MRI or fMRI) for early diagnostics of AD research (11–14). Meanwhile, some recent studies show an upgrade from uni-model to multi-model research involving two or more image modalities (MRI, fMRI, Pet etc.) (15–17) and behavioral information from neuropsychological tests (18). However, integration of respective modulation of neuro-chemicals with the imaging information as well as neuropsychological scores has never resulted in a correlation. Hence, there is an urgent need to unify the data diversity for early diagnostic biomarkers for AD.

Big data collections are combinations of multi-modal datasets that are individually manageable, but—as a group—are too large to handle seamlessly and accurately using a single machine. With the growth in data generation, ML faces the challenge of efficiently processing and learning from big data. In this context, the development of advanced tools involving big data analytics (BDA) is the current need for handling enormous volumes of diverse data, which grow with extraordinary velocity. A more comprehensive approach is required that can accommodate the velocity, volume and variety (19) in AD research. “Volume” (19) of data, the first characteristic, has been increasing because of the available size and diversity of heterogeneous data acquired using multi-modalities (e.g., MRI, MRS, and neuropsychological outcomes) with definite protocols. The second big data characteristic, i.e., “Variety” (19), relates to the heterogeneous nature of data generated from diverse data sources. Continuously growing data with exponential and high processing speed implicates “Velocity” (19) as the third important characteristic of the BDA system. As the data volume and variety are constantly increasing there is a need to store and process a large amount of diverse data. Therefore, a popular ecosystem, “Hadoop,” is being developed which offers distributed storage and processing at large-scale and is fast and accurate. Hadoop itself presently contains four modules named as follows: “Hadoop common,” which supports the other Hadoop modules; “Hadoop Distributed File System” (HDFS), which provide distributed storage; “Hadoop Yet Another Resource Negotiator” (YARN), for job scheduling and cluster resource management; and “Hadoop MapReduce,” for parallel processing of large data sets (20).

Comprehending the big data challenges in AD research (21), a new and specific Hadoop (20)-based platform is proposed which incorporates clinical data management, processing, and analysis of the diverse multi-modal imaging, neuro-chemical, and neuropsychological data. This perspective focuses on the available big imaging data generated from multiple modalities such as MRI, MRS, and neuropsychological tests and addresses the current research challenges with possible solutions on the development of a dedicated big data framework for AD research. The proposed novel scheme is a first step toward observation of a new research direction by combining the anatomical, metabolic and cognitive changes, which can provide better understanding of the early onset and progression of AD.

## Big Data Challenges in AD Research

Extensive research has been accomplished over the past few decades in the domain of ML for big data. But challenges remain inherent, some of which include the followings:

- *Large data size:* A major challenge of big data research in AD is to collect, store and standardize the large size of diversified and complex heterogeneous data from distributed sources for further processing and to analyze them at a high velocity. The captured distributed data from different data sources require common and standardized data acquisition protocols, data nomenclature, and data sharing standards for further processing.
- *Feature extraction:* High dimensionality is a common characteristic of big data, especially when using multi-modalities. Feature extraction is used to reduce the dimensionality of data, extracting information that is useful for classification. To date, limited literature is available on extracting features from multiple modalities. Feature selection for the reduction of dimensionality can be achieved using principal component analysis and other similar techniques.
- *Classification:* Selecting the classifiers for specific modality is also a challenging task. Hence, validation for proper benchmark classifier is essential.
- *Noise and missing values:* Sometimes, MRI images, and MRS signals are noisy or contain artifacts. Quality checks should be performed to identify, evaluate, and discard the data from the analysis pipeline. Also, neuropsychological data may contain some missing values. Inclusion of noisy data and missing values may lead to inaccurate models or may lead to overfitting.
- *Security:* Another challenge at a global level for AD research exists for data sharing and security. Data sharing standards should be strictly followed at every level.

## Proposed Big Data Analytics (BDA) Framework

**Figure 1:**
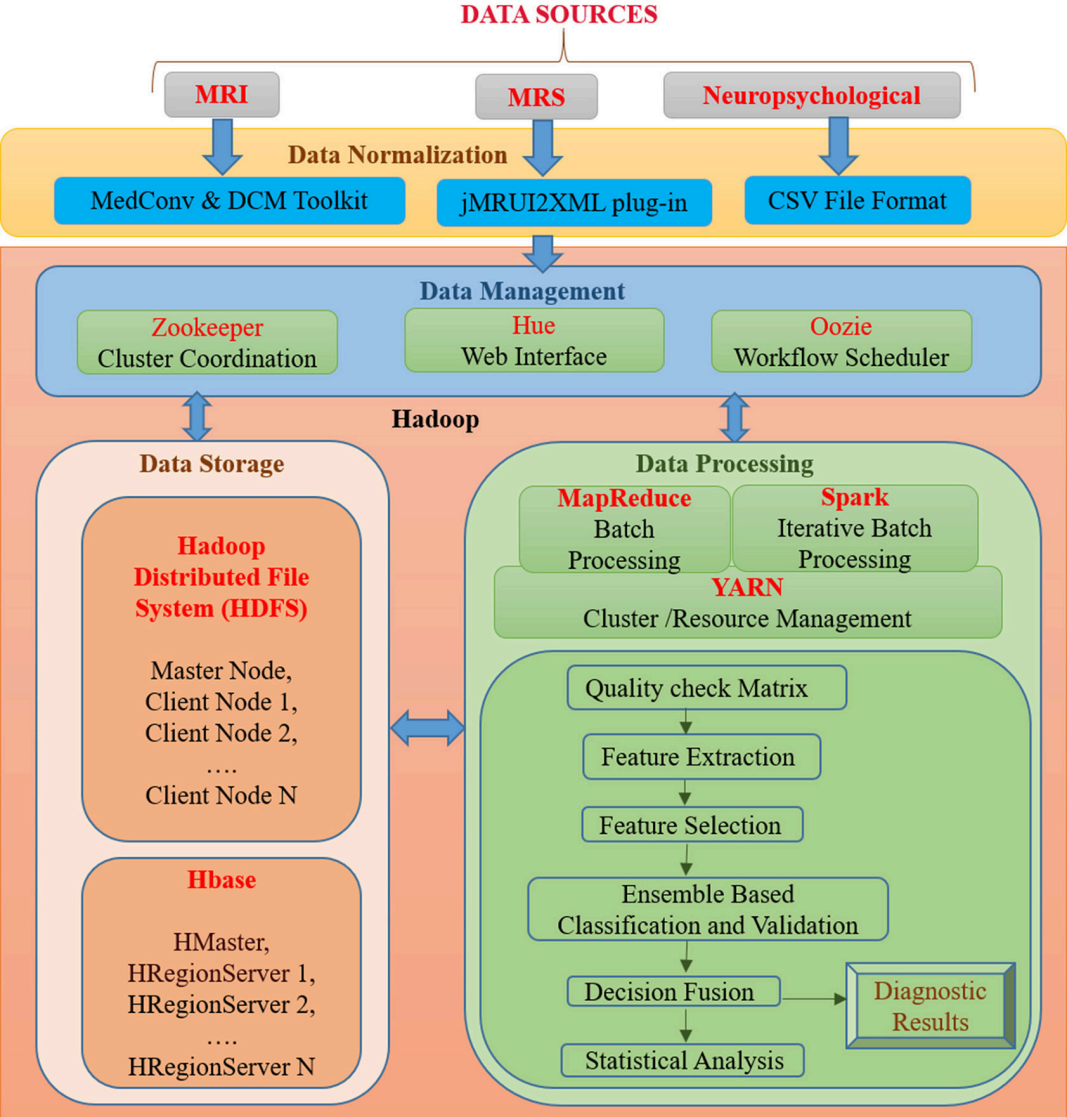
An integrated framework for Big Data Analytics (BHARAT) using Hadoop with four components: Data Normalization, Data Management, Data Storage, and Data Processing. Data Normalization includes conversion of raw data into a suitable format for further processing, which is collected from diverse data sources. Data Management deals with organization and management of data from diverse sources. For example, Zookeeper helps in maintaining synchronization among distributed sources and Hue supports querying and visualization of data. Data storage consists of the HDFS file storage system to store a large amount of data and HBase is a NoSQL database used to read/write data in real time. Data Processing component contains different packages and libraries for processing and analysis of data from different modalities. It performs feature extraction, selection, classification, and decision fusion for accurate classification of data followed by statistical analysis. Diagnostic results are provided as final outcomes and will be further verified from clinician.

BDA framework integrates structured and unstructured data organization, storage, processing, and analyzing a vast volume of complex data. It utilizes data organization, parallel computing, distributed storage techniques, and ML-based algorithms that can deliver fast and scalable data processing. A proposed BDA framework for multi-modal data to classify between healthy old (HO), mild cognitive impairment (MCI), and AD is shown in Figure 1. The proposed framework can be broadly partitioned into four major components, namely (1) Data Normalization, (2) Data Management, (3) Data Storage, and (4) Data Processing. This section discusses the details of each of the four components of the proposed Hadoop-based BDA framework for AD classification and its progression. The framework facilitates accommodation of a massive amount of heterogeneous data followed by data-specific pre-processing, analysis of processed data outcomes, and inference of diagnostic results.

### Data Normalization

In the proposed BDA framework, multi-modal (MRI, MRS, and neuropsychological) data originating from distributed sources are ingested in a single platform. The data normalization component deals with the organization of heterogeneous multi-modal neuroimaging data acquired from different modalities, which requires interfaces to accommodate the diversified data in a single platform. In the proposed framework, MRI DICOM images are used. Therefore, in the case of neuroimaging informatics technology initiative (nifti) MRI images, MedCon (22) is used for medical image conversion. DICOM Toolkit (DCMTK) (23) is an assortment of libraries and applications implementing the DICOM standards. It comprises of software for examining, constructing, sending and receiving images over a network connection. For MRS data processing, the jMRUI2XML (24) plugin is used to process the MRS data and then export it to XML format, which can be used for further processing. Neuropsychological scores are uploaded into the file system in the Comma-Separated Values (CSV) file format usable for processing.

### Data Management

The data management component includes tools for organization and user interaction using a front-end and back-end system. Front-end refers to the interface for direct user interaction and accessibility of the system by way of the back end. Along with the front end, the back end deals with storage of raw as well as processed data. It also implements responses to the front end. Front-end functionality consists of the Hadoop user experience (Hue)^1^, Apache Zookeeper (25) and Oozie (26) for interface, coordination and scheduling. Hadoop user experience (HUE) provides a web interface for Hadoop for accessing, querying, and visualizing data. This interface stands between a large amount of warehouse and other tools such as HBase, YARN, Oozieetc. It features file browsers for HBase (27) and HDFS (28), and a job browser for YARN (29). It works in coordination with Oozie (26), YARN (29), HBase, HDFS, and many other big data tools. Zookeeper helps in maintaining synchronization among distributed sources and maintains configuration information. It is also able to handle partial network failures (25). Oozie is a workflow scheduler for Hadoop jobs that specifies a sequence of operations and coordinates between them to complete the job (26). Back-end functionality consists of the HDFS and HBase. The front and back ends jointly support in developing a complete framework composed of a web interface for input from diverse data sources and user interaction followed by storing input, processing and analyzing it.

### Data Storage

The data storage component is essential for organizing structured and unstructured data acquired from different modalities. Storage facility is provided by both HDFS and HBase. Hadoop Distributed File System (HDFS) is developed to store a large amount of data across different nodes of commodity hardware. It has master-slave architecture comprised of data nodes (slave node) wherein each stores blocks of data, fetches upon requirement, and acknowledges back to the name node (master node) (28). Metadata (data about data) storage is also a critical element for storage. In the data node actual data is stored and the name node stores the metadata, including file location, block size, file permission, etc. In case of any node failure, it has built-in fault tolerance mechanism. The main drawback of HDFS is that it operates on a write once read many (WORM) pattern. Therefore, if changes are required even on a single data point, the whole file must be rewritten. HBase is a non-relational, referred to as Not only SQL (NoSQL), database that provides quick random access to a large amount of structured data. HBase also supports structured, unstructured as well as semi-structured data. Data in HBase is stored in columns by sorting them according to key value pairs. It also contains cryptographic software to provide data security. HBase provides a library and runtime environment within the HBase region server and master processes for executing user code (30).

### Data Processing

Data processing includes quality checks, feature extraction, selection, and decision incorporation. Subsequently, these features are used for classification of subjects into HO, MCI, and AD; followed by statistical analysis and verification. Each component of this layer is further discussed below.

#### MapReduce, Spark, and YARN

The proposed framework classifies subject categories between HO, MCI, and AD using data from three different modalities. Such high-dimensional datasets have problems with storage, analysis, and visualization. MapReduce is a reliable and fault-tolerant framework used to process large amounts of data in parallel on large clusters (31). MapReduce has two functional phases: Map and Reduce. Map organizes raw data into key-value pairs and Reduce processes data in parallel. Apache Spark's MLlib machine learning library is used for feature extraction, dimensionality reduction, classification, and basic statistics (32). MLlib relies on iterative batch processing of Spark. Spark supports iterative processing and improves speed by utilizing in-memory computation (33). YARN helps in job-scheduling and cluster resource management. It also handles and schedules resource requests from the client to run an application and helps in executing the process.

#### Quality Check Matrix

The quality of the data check was performed before starting the processing of multi-modality data. Further, the framework also takes care of missing values from data to avoid overfitting.

#### Feature Extraction

Feature extraction is used to extract distinctive and disease-specific features from the image. For MRI data, structural statistical features will be extracted which consist of statistical information from regions of interest (ROI) specific to the AD disease process for AD patients. Statistical features include entropy, histogram-based features like mean and median, texture information of AD-related brain regions like the hippocampus, frontal cortices, etc. In MRS, spectral features representing metabolic information of ROI are extracted in the form of neurochemical content peak area. Neuropsychological data contain scores like MMSE (6), CDR, GDS-SF (9), HIS (8), FAQ (7), and TMT-A and TMT-B (10).

#### Feature Selection

Features extracted from MRI, MRS, and neuropsychological data are still high-dimensional data for classification. Principle component analysis (PCA) (34) from the ML library is used to reduce the dimension of features by obtaining the set of principal values. The idea of PCA can be extended to high-dimensional space for maximizing variance using the kernel trick.

#### Ensemble Based Classification and Decision Fusion

Our goal for using multi-modality is to improve the accuracy of the classification of AD patients compared to the decision made by using only one source of data. Therefore, ensemble-based classifiers and their decision fusion approach are proposed for accurate automated classification (35). The concept of using the ensemble approach for data fusion is that a separate classifier is used to train each modality that comes from different source. Further, a decision made by an individual classifier will be combined according to the appropriate combination rule. We can use the sum rule to obtain data fusion to improve the diagnostic performance.

#### Statistical Verification

The accuracy of disease diagnosis is important as it has a direct impact on patient treatment. Therefore, statistical analysis of classification accuracy will be conducted to validate the results of the proposed framework. Parameters for statistical analysis include sensitivity, specificity, classification accuracy, and receiver operating characteristics (ROC) (36–38). Diagnostic results are verified from clinicians.

## Discussion

To date, AD pathogenesis and effective diagnostic intervention remain unclear. However, it is believed that the available solution is to control its progression from MCI to AD. Early diagnostic biomarkers originating from the combined analysis of the information derived from multi-modal data (MRI, MRS, and neuropsychological) can provide insights to the actual cause of AD. This will finally lead to therapeutic interventions to be followed by clinical trials.

To identify diagnostic AD biomarkers for a large amount of diversified data with the help of accurate feature extraction and classification, a new multi-modal BDA system framework is now proposed. The present BDA framework is designed for collecting, storing, organizing, and analyzing the multi-modal big data.

Big data framework with Hadoop provides an integration of various modalities at one platform. The proposed BDA framework facilitates to normalize and preprocess raw MRI, MRS, and neuropsych data into a suitable format that can be used for further processing. The pre-processed big data will be managed and stored using Hadoop. For improving speed and resource management, the MapReduce programming model and YARN resource manager will be used. Classification of subjects into specific class (HO, MCI, and AD) using neurochemical information from MRS data along with anatomical details of MRI data and neuropsychological data, is the main goal of this study. Feature extraction from a distinct modality is an important step; therefore, new feature extraction techniques are proposed for MRI as well MRS data. For dimensionality reduction of the extracted features, variants of PCA are proposed which contain maximum variance among features. For each modality a different classifier is used, and the decision of each classifier will be fused to get final diagnostic results. Statistical analysis is performed with Spark libraries for validation (39, 40).

Our focus is not only on best classification performance using multi-modality data, but also to determine complimentary information of these modalities with an ensemble-based classifier.

We would like to emphasize that BDA for early diagnostics from various modalities at present are only based on MRI images of patients. The neurochemical-like antioxidant glutathione depletion analysis from brain hippocampal regions are extremely sensitive and specific, with more than 92% sensitivity and 94% specificity (5). This perspective brings these novel features to be included, which are close to the disease process and present a realistic approach.

We would also like to indicate that alternative approaches are available. There are equally promising BDA in Alzheimer's disease: Google BigQuery, Presto, Hydra, and Pachyderm. These alternative platforms compared to Hadoop hold huge promise in BDA for Alzheimer's disease in the coming years. To the best of our knowledge, we have not come across any manuscript where this type of scheme is proposed. We are aware that various research groups have started working in this important and challenging area.

We have already developed a prototype based on the scheme presented in Figure 1 and it is operational for actual data analysis on a smaller scale. We have tested this scheme involving 128 MRI images, 128 MRS data points, and 128 neuro-psychological data points (scores) in a Scalable Hadoop cluster consisting of two nodes with 36 + 2 cores. The operation takes around 5 min to process these pilot data (MRI, MRS, and neuropsychological) in the PySpark toolbox using different deep-learning libraries along with tensor flow.

## Conclusions

Large-scale data analysis using brain imaging, metabolic, and neuropsychological scores provides information about disease progression and identifies early diagnostic biomarkers. Hence, conceptualization of BIG analytics using three critical points of information is an important step and it will likely to provide much contribution in the development of a working BDA framework, where medical physicists, clinicians, and engineers will work hand-in-hand to advance an effective tool for early diagnosis or prediction of AD.

## Author Contributions

PKM conceptualized the idea, wrote the manuscript, contributed to the discussion, literature search, and figure modification, and proofread the manuscript. AS was involved in writing the manuscript, literature search, figure preparation and modification, participated in discussion, and proofread of the manuscript. DS and TG contributed equally to the literature search, manuscript preparation, participated in discussion, figure preparation, modification, and proofread the manuscript.

## Dedication

PKM dedicates this manuscript in honor of his parents (Mr. Bhadreswar Mandal & Mrs. Kalpana Mandal) and volunteers (the elderly, MCI and AD patients who participated in various research studies in the NINS laboratory).

### Conflict of Interest Statement

The authors declare that the research was conducted in the absence of any commercial or financial relationships that could be construed as a potential conflict of interest.

## Footnotes

^1^http://gethue.com/
